# Chemically Bonded Phases for the Analysis of Trace Amounts of Organic Pollutants

**DOI:** 10.1080/15376510701623938

**Published:** 2008-06-23

**Authors:** I. Rykowska, W. Wasiak, A. Szymański, K. Szyrwińska, J. Lulek

**Affiliations:** Department of Analytical Chemistry, Faculty of Chemistry, Adam Mickiewicz University, Grunwaldzka 6, 60-780, Poznan, Poland; Department of Inorganic and Analytical Chemistry, Poznan University of Medical Sciences, Grunwaldzka 6, 60-780, Poznań, Poland

**Keywords:** Bisphenol A (BPA), Gas Chromatography, Solid Phase Extraction (SPE)

## Abstract

This work describes some results of identification and determination of bisphenol A (BPA) in powdered milk by applying the gas chromatography. To determine BPA contents in the milk and to reduce the matrix interference associated with the constituents of the powdered milk, we performed the following activities. First, we ultra-centrifuged the dissolved milk solutions. Next, we preconcentrated the analyte in the supernatant using a C_18_ and new sorbent with chemically bonded ketoimine group solid phase extraction column. Finally, we used gas chromatography for the determination of BPA in the samples under study. A recovery of bisphenol A from spiked milk samples was also performed, with recovery result located at 91% ± 3%/94% ± 2%.

## INTRODUCTION

Some recent research has shown an influence of many chemical substances, so far treated as of no importance, to the health of humans and animals. Such chemicals as natural and synthetic hormones, metalorganic compounds, persistent organic compounds, monomers, and some chemical additions used in the plastic industry are able to disturb the natural hormonal balance of the human body, as well as to cause several diseases both for humans and animals (Toft et al. 2004; LaKind 2004; Brent and Weitzman 2004).

Recently, bisphenol A (BPA) has become a compound of particular interest of ecotoxicologists (Zoeller 2005; Saale et al. 2005; Ikezuki et al. 2002; Schönfelder et al. 2002). This compound has been produced and widely used incessantly since 1905. The annual amount of production of BPA is estimated to be millions of tons (Staples et al. 1998). BPA is used for many industry activities, including a synthesis of polycarbonate plastics, epoxy resins, and polyacrylates. These plastic materials are used for the production of (among others) baby bottles and internal coating layers for the packings for the baby food industry, such as powdered milk and milk mixtures (Schönfelder et al. 2002; Staples et al. 1998; Biles 1997; Kuo and Ding 2004).

For many years, BPA was seen as a compound that is not dangerous for the environment and living organisms, mainly due to its relatively low half-life time (a few days in the water) and low value of the partition coefficients octanol/water (lg_o/w_, 3.8) (Staples et al. 1998). However, by the end of the 20th century, BPA was detected in the environment, in drinking water, and in food packages. After some research, BPA was classified by the European Commission as “an external derivative having an adverse influence on human health and offspring.” Compared with natural fitoestrogenes, estimated daily intake of BPA is quite low (1 ÷ 6.6 μg/kg body weight/day) (Zoeller 2005; Greim 2004). Recently, many publications confirmed estrogenic activities of BPA in vivo, even taken in very little doses (Ikezuki et al. 2002; Schönfelder et al. 2002). However, the assessment of harmful effects of hormonally active compounds such as BPA (Zoeller 2005; Saale et al. 2005; Staples et al. 1998; Greim 2004) has not been unambiguous so far (Saale et al. 2005).

Fetuses and neonates are the group of particular sensitivity to the *endocrine disrupter* substances, such as BPA. As recently published, BPA crosses the placenta, exposing the vulnerable fetus to BPA circulating in the mother's blood. After birth, the infant is additionally exposed to BPA presented in human milk (Schönfelder et al. 2002; Kurodawa et al. 2003; Yoshimura et al. 2002; Hong et al. 2004; Matsumoto et al. 2004). It was proven by the measurement and comparison of BPA levels in blood serum of mothers, fetuses, and newborn babies (umbilical blood serum).

The main way of exposure to BPA by breast-fed and bottle-fed infants is related to human milk or dietary supplements such as powdered milk. Sun et al. (2004) and Otaka et al. (2003) informed that the level of BPA in the milk of Japanese women varies from 0.61 to 0.65 ng/mL^−1^ of milk. These values are much lower in comparison with the BPA levels in five different dietary supplements for newborn babies (44 ÷ 115 ng/g^−1^ of milk), as reported by Kuo and Ding (2004). Further research and determination of the BPA levels in breast milk as well as food products for the infants is necessary.

This work is concentrated on determination of BPA in the milk and milk derivative products. The main goal of this work was to propose optimum conditions for the isolation and further determination of BPA in the milk samples, by the use of solid phase extraction (SPE), gas chromatography (GC) coupled with flame ionization detection (FID), and low-resolution mass spectrometry (LRMS).

## EXPERIMENTAL

### Materials

**FIGURE 1:**
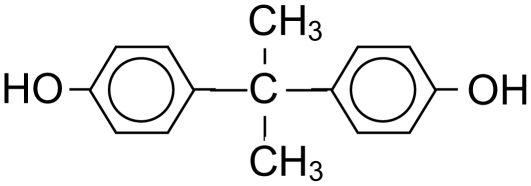
The structure of 2,2-bis-(4-hydroxyphenyl)-propane (BPA).

BPA was purchased from Sigma-Aldrich, and had a purity of equal to or greater than 98%. The structure of the bisphenol A is shown in Figure 1.

All standard stock solutions were prepared in methanol and used after proper dilution with the same solvent. The water was purified in the Milli-Q apparatus (Millipore S.A. 67120 Mol-sheim, France). BSTFA (bis-trimethylylsililtrifluoroacetamide) with 1% TMCS (trimethylchlorosilan) was purchased from Sigma-Aldrich and Silica gel (Baker Analyzed) from J.T. Baker.

### Apparatus

Elemental analysis was performed on 2400 CHN Elemental Analyzer (Perkin-Elmer, Norfolk, USA), while NMR spectra for the solid phases (^29^Si CP MAS NMR) were taken using spectrometer 300 MSL (Bruker, Rhenstteten, Germany).

The chromatographic separation was performed using the following hardware:

- Gas chromatography VARIAN CP-3380 equipped with FID. A CP-SIL 5 CB (30 m x 0.32 mm; DF = 0.25 μm) capillary column was used. Temperature program: 2 min at 100°C, then programmed at 10°C/min^−l^ to 280°C and held for 17 min; injector temperature: 300°C; detector temperature: 300°C. Helium was the carrier gas. All the work was carried out in a constant flow mode set at 3.5 mL/min^−1^.
- Gas chromatography Perkin Elmer AUTOSYSTEM XL TURBO MASS equipped with LRMS. A DB-5 (30 m x 0.25 mm x 0.25 μm) capillary column was used. Temperature program: 2 min at 150°C, then programmed at 30°C/min^−1^ to 270°C and held for 10 min; injector temperature: 250°C. The MS acquisition parameters were: ion source 300°C; electron ionization 70 eV. Dwell times were set at 0.1. Full scan spectra were run in the electron impact (EI) mode from m/z 100 to 500. Besides, two ions were monitored in EI selected ion monitoring mode (SIM). These ions were 213 and 228 for BPA and 357 and 372 for derivatization product of BPA with BSTFA.

### Methods

To determine BPA content in the milk and to reduce the matrix interference associated with the constituents of the powdered milk, we performed the following analytical procedure. First, we ultra-centrifuged the dissolved milk solutions. Second, we preconcentrated the analytes in the supernatant by the SPE technique, using a C_18_ and the new sorbent with chemically bonded ketoimine groups for solid phase extraction column. Finally, we used gas chromatography for the determination of BPA in the samples under study. To this goal, we applied gas chromatography with FID and LRMS.

#### Sorbent preparation

**FIGURE 2:**
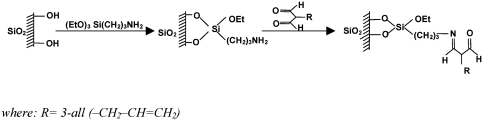
A scheme of the modification procedure for the packings.

The scheme of the new sorbent preparation is shown in Figure 2. Five grams of dry silica was immersed in a mixture of anhydrous xylene and 3-aminopropyltriethoxysilane. The mixture was boiled for 12 h in a vessel equipped with a reflux condenser. The contents were continuously stirred and carefully protected against the moisture. Unreacted silane was extracted with xylene and hexane in a Soxhlet apparatus. After that it was dried under vacuum and finally subjected to the so-called “end capping” reaction with hexamethyldisilazane in order to deactivate free silanol groups remaining at its surface.

The second step was bonding of amino groups using an appropriate derivative of 3-pentano-2,4-dione. As previously states, reaction was performed under continuous stirring in anhydrous xylene and lasted 12 h. The system was protected against the moisture. The final product was extracted subsequently with xylene and hexane in a Soxhlet apparatus. Finally, the modified silica was dried under vacuum.

#### Sample Powder Milk Preparation

The powdered milk samples were purchased from Polish supermarkets. An accurately weighed sample of 0.5 g of the analyzed milk was dissolved in 5 mL 50% (v/v) of ethanol solution, and then mixed for 2 min in an ultrasonic chamber. Such prepared sample was centrifuged for 40 min at 5000 rpm, and finally filtered through a membrane filter 3W.

Before extraction, each SPE cartridge was conditioned with 5 mL of methanol and 15 mL of deionized water on an SPE manifold. The accurately weighed milk sample mentioned above was introduced on top of conditioned SPE column. Once the total amount of a sample was put on, the sorbent was dried for 10 min under vacuum, and the preconcentrated BPA was eluted by the use of 3 mL of methanol. The extract was concentrated or dried up and further injected to GC-FID chromatograph or derivatized by the silylating agent, respectively.

#### Derivatization Procedures of Sample Powder Milk

The evaporated extract was derivatized by adding 100 μL of the silylating agent containing BSTFA (bis-trimethylylsililtrifluoroacetamide) and 1% TMCS (trimethylchlorosilan). The vial was vortex mixed and heated at 80°C for 30 min. After cooling, the derivatized solution was evaporated to dryness, and the residue was redissolved in 100 μL of chloroform. One microliter of derivatized milk extract was injected into the GC system. The derivatives of analytes were ready for GC/FID and/or GC-MS analysis (Kuo and Ding 2004).

#### GC Analysis

The BPA identification was performed by GC-FID and GC-LRMS methods, using the data from chromatograms. BPA was identified by a comparison of the retention times (RTs) of the peaks from calibration standard before and after derivatization with peaks from nonderivatized and derivatized cleaned-up extracts of milk. Additionally, a confirmation of BPA presence in milk extracts was executed by a comparison of corresponding mass spectra of the peaks on the chromatograms mentioned above.

Quantitative measurements of BPA in spiked and nonspiked powered milk samples were carried out using peaks areas.

## RESULTS

To investigate physicochemical properties of the obtained packings, these packings were subjected to elemental analysis. One determined the contents of carbon, hydrogen, and nitrogen and estimated the specific surface area. The surface concentration of bonded siloxane molecules (denoted by α) in μmol/m^−2^ was calculated from the carbon content according to the following equation:

$$\alpha =\frac{\%C\cdot 10^{6}}{(100\cdot n\cdot 12-\%C\cdot M)\cdot S_{\text{BET}}},$$

where %*C* denotes percent of carbon contribution, *n* the number of carbon atoms in the molecule of bonded silane, *M* the molecular mass of the siloxane, and *S*_*BET*_ the specific surface area [m^2^/g^−1^]. The obtained results are presented in Table 1.

**TABLE 1:** Physicochemical properties of the sorbents under study

	Elemental analysis,%						
					
Sorbent	C	H	N	m^2^/g^−1^①	μmol/m^−2^②	nm③	ml/g^−1^④
Silica gel	0.05	0.99	—	552	—	6.18	0.88
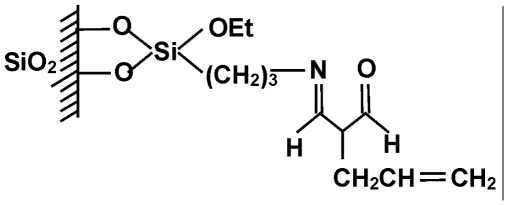							
	13.44	3.34	1.96	326	4.37	4.87	0.41

① Surface area.

② Surface concentration of silane.

③ Pore diameter.

④ Specific pore volume.

**FIGURE 3:**
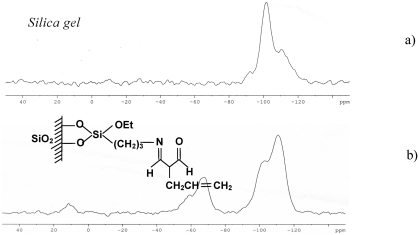
Spectra ^29^Si CP MAS NMR of (a) the unmodified silica and (b) the sorbent under study.

Spectra ^29^Si CP MAS NMR of the unmodified silica are presented in Figure 3a, while the spectra of the sorbent under study are presented in Figure 3b. An analysis of these spectra confirms that a reaction of the synthesis took place at the silica surface. During this reaction, the geminal silanols were blocked. The distinct signal at −90 ppm for the spectrum of the unmodified silica is definitely weaker for the modified silica. At the same time, the signal at −100 ppm is changed, and this fact proves that the isolated silanols are blocked as well. A clear signal at +12 ppm accompanied by the signals at −46 ppm (T_1_), −50 ppm (T_2_), and −64 to −70 ppm (T_4_ + T_4_′) points out the modification of the silica by trifunctional silane and the“end capping”process by the use of hexamethyldisilazane.

## DISCUSSION

### Identification of BPA in Powdered Milk Extracts

The criteria of identification of BPA in analyzed milk extracts were following:

- The retention times of the peaks on GC-FID and GC-MS chromatograms of milk extracts should correspond to those on the chromatograms of BPA standard solution.
- The retention times of the peaks on GC-FID and GC-MS chromatograms of derivated milk extracts should correspond to those on the chromatograms of derivated standard solution of BPA.

**FIGURE 4:**
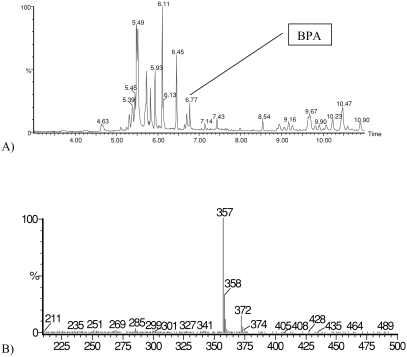
Typical chromatogram of the total ion current TIC (100–500 amu), taken for the powdered milk extract (A), and (B) corresponding mass spectrum of BPA.

Typical chromatogram of the total ion current, in the range of the monitored mass 100 to 500 amu, is shown in Figure 4A. In Figure 4B, the mass spectrum of the peak corresponding to the peak of BPA is shown.

To establish the retention time of BPA, average retention time was computed based on the data from six GC/FID and GC/MS chromatograms, as well as standard deviation (SD) of the average retention times was calculated. The obtained results are shown in Table 2.

**TABLE 2:** Retention parameters of BPA using various GC detectors

	Retention time RT ± SD (min) (n = 6)	
	
Detector	BPA standard solution	BPA + BSTFA
MS	6.727 ± 0.013	6.757 ± 0.006
FID	13.199 ± 0.004	13.805 ± 0.004

The repeatability of the RTs calculated from six replicate analyses of a standard BPA solution as well as the product of standard BPA derivatization was 0.19, 0.09% (MS) and 0.03, 0.03% (FID), respectively.

### Limit of Detection (LOD) and Quantification (LOQ)

The limit of detection (signal-to-noise ratio = 3) and quantification limit (signal-to-noise ratio = 10) for BPA was established only for the GC-FID method. This method limit of detection of BPA is approximately 140 ng/g^−1^ of milk, while the method limit of quantification is equal to 379 ng/g^−1^ of milk.

### Recovery Study

**FIGURE 5:**
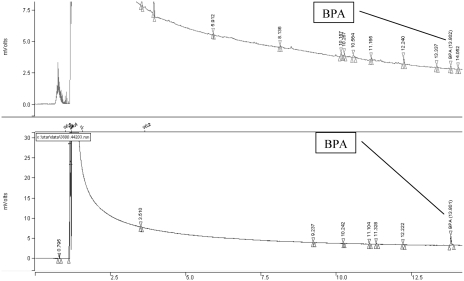
GC/FID chromatograms of powdered milk samples: (a) not spiked, (b) spiked with BPA.

Recovery tests were performed for the powdered milk samples spiked with known amount of BPA (0.5 μg/g^−1^). These tests were performed in triplicate, using the above described method, with the recovery result obtained for the C_18_ sorbent equal to 91% ± 3% and that for the sorbent with chemically bonded ketoimino groups equal to 94% ± 2%. The typical GC-FID chromatograms of nonspiked and spiked milk samples are presented in Figure 5.

### Quantification of BPA in Powdered Milk Samples

BPA determination in milk extracts was performed according to the procedure described in the Experimental section. The method of standard addition was used in quantitative analysis of BPA in milk extracts. Dependencies between the peak area and BPA concentration added to milk samples were determined. Based on these dependencies, the calibration graphs were prepared. To this goal, some pattern solutions were used with the BPA concentration ranging from 0.5 to 50 μg/cm^−3^. The final peak area was taken as an average of three experiments in turn. Calibration plot was described with the general equation: *y* = *ax* + *b*, where *y* is the peak area, and *x* the amount of determination compound in μg/ml^−1^. For the observed range of concentration values, a linear correlation is observed of the calibration curves for calibration coefficients greater than 0.9997.

The results of BPA determination (GC/FID) in the powdered milk are presented in Table 3.

**TABLE 3:** BPA level determined in the powdered milk samples, using two various SPE sorbents

	Concentration of BPA in a sample ng/g^−1^ milk ± SD	
		
Milk sample	C_18_	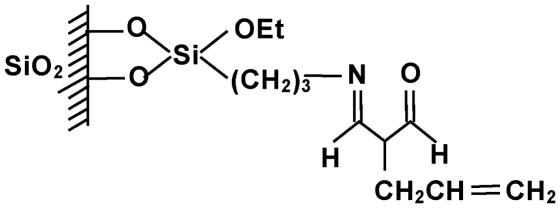
I	182 ± 37	191 ± 11
II	272 ± 15	273 ± 9

## CONCLUSIONS

As proven by our experiments, newly synthesized sorbent with chemically bonded ketoimine groups may be used for preconcentration of BPA by the use of the SPE method. The proposed analytical procedure of BPA determination in powdered milk seems to be sufficiently sensitive and selective. A combination of the SPE and GC make possible the determination of this compound at a level of ng/g^−1^. The proposed method of extraction and preconcentration of the analyte is characterized by a high amount of recovery 91% 94%.

The measured concentration of BPA in the powdered milk belongs in the range of 182 to 273 ng/g^−1^ milk and is comparable to those obtained by other authors (Kuo and Ding 2004). Our experiment proved that the powered milk can be one of the sources of exposure of bottle-fed infants to BPA.
